# Platelet lysate outperforms FCS and human serum for co-culture of primary human macrophages and hMSCs

**DOI:** 10.1038/s41598-019-40190-9

**Published:** 2019-03-05

**Authors:** Tina Tylek, Tatjana Schilling, Katrin Schlegelmilch, Maximilian Ries, Maximilian Rudert, Franz Jakob, Jürgen Groll

**Affiliations:** 1 Department for Functional Materials in Medicine and Dentistry and Bavarian Polymer Institute, University of Würzburg, Würzburg, Germany; 2 Department of Orthopedics, Orthopedic Center for Musculoskeletal Research, University of Würzburg, Würzburg, Germany

## Abstract

*In vitro* co-cultures of different primary human cell types are pivotal for the testing and evaluation of biomaterials under conditions that are closer to the human *in vivo* situation. Especially co-cultures of macrophages and mesenchymal stem cells (MSCs) are of interest, as they are both present and involved in tissue regeneration and inflammatory reactions and play crucial roles in the immediate inflammatory reactions and the onset of regenerative processes, thus reflecting the decisive early phase of biomaterial contact with the host. A co-culture system of these cell types might thus allow for the assessment of the biocompatibility of biomaterials. The establishment of such a co-culture is challenging due to the different *in vitro* cell culture conditions. For human macrophages, medium is usually supplemented with human serum (hS), whereas hMSC culture is mostly performed using fetal calf serum (FCS), and these conditions are disadvantageous for the respective other cell type. We demonstrate that human platelet lysate (hPL) can replace hS in macrophage cultivation and appears to be the best option for co-cultivation of human macrophages with hMSCs. In contrast to FCS and hS, hPL maintained the phenotype of both cell types, comparable to that of their respective standard culture serum, as well as the percentage of each cell population. Moreover, the expression profile and phagocytosis activity of macrophages was similar to hS.

## Introduction

Monocytes and macrophages evolving thereof are cells of the innate immunity, which can be found in most tissues of the human body^1^. Macrophages are highly plastic cells, which can change their polarization in response to various signals. Thus, several classes could be described based on their expression and phenotypical profile^2,3^. The two main sub-populations are the classical (M1)-or alternative (M2) activated macrophages^4^. The M1 type is characterized by the expression of proinflammatory cytokines, like IL-1β, IL-6, IL-8 or TNF-α, microbicidal activity and the production of reactive nitrogen and oxygen intermediates^5^. In contrast, M2 macrophages are mainly involved in tissue remodeling and immunoregulatory processes. In addition, they show a high phagocytic activity, the expression of scavenging receptors, e.g., CD163, mannose (CD206) receptors or anti-inflammatory cytokines like IL-10^6^. During *in vitro* studies, it is also possible to differentiate macrophages spontaneously, i.e. without the addition of differentiation factors, into the so-called M0 type only by environmental factors like specific materials or due to co-cultivation with other cell types^7^.

Cells that tightly interact with macrophages *in vivo* are mesenchymal stromal cells (MSCs)^8–10^. These adult multipotent stem cells can differentiate into various cell types, like osteoblasts^11^, chondrocytes^12^ and adipocytes^13^. They are known for switching the phenotype of proinflammatory macrophages into the anti-inflammatory M2 type, and thus are part of tissue regeneration and wound healing processes together with the latter^14–16^. Accordingly, the crosstalk of both cell types is often analyzed in biomaterial research. Here, the special interest is to investigate, whether there are any changes in macrophage polarization or MSCs differentiation in *in vitro* as well as *in vivo* studies depending on the designed biomaterial^10,17–19^, eventually with the aim to evolve design criteria for biomaterials to favor or even provoke a healing response after implantation.

So far the majority of *in vitro* studies which examine the interaction of either hMSCs or macrophages with biomaterials rely on cell monoculture. One issue hindering the establishment of an *in vitro* co-culture especially of these both cell types is the cultivation in different culture media. While human monocytes and macrophages are most commonly cultivated in RPMI-1640 with human serum^7,16,20^, hMSCs are usually cultivated in MEM-based media supplemented with fetal calf serum (FCS)^21–23^. Recent studies have already shown that hMSCs can also be cultivated in medium with human platelet lysate (hPL) instead of FCS, without the loss of their differentiation potential and immunomodulatory effects^24–26^.

PL can be prepared via freeze-thaw cycles of platelets and subsequent centrifugal separation of the debris from all the bioactive platelet factors^27,28^. These include for example the platelet-derived growth factor (PDGF), transforming growth factor (TGF)-b1, vascular endothelial growth factors (VEGF), epidermal growth factor (EGF), attachment factors, and enzymes^29^. Like macrophages and hMSCs, platelets are part of tissue regeneration processes^30^. While platelets coagulate and degranulate upon wounding, they release bioactive factors. These lead to inflammation and thus neutrophil and macrophage activation, fibroblast, smooth muscle cell, and MSC recruitment as well as collagen synthesis and angiogenesis, resulting in tissue regeneration^24^.

Hence, especially for *in vitro* studies in the field of tissue regeneration and biomaterial research, the use of platelet lysate could mimic the natural environment of an *in vivo* situation better than other commonly used supplements and showed beneficial effects, if incorporated in biomaterials^31,32^. When PL is used as a serum supplement, it is, however, often described that heparin has to be added to prevent the coagulation of the medium in cell cultures^33^. However, heparin is also known to stimulate macrophage polarization^34,35^, which is unfavorable for the analysis of their spontaneous differentiation. In the present study, we thus investigated the use of PL as an alternative for macrophage *in vitro* cultivation as well as for co-culture experiments with hMSCs. Furthermore, we analyzed the need of heparin as a substitute in both culture systems.

## Results

### Macrophage cultivation in RPMI-1640 medium with different sera

#### Influence of different sera on cellular phenotype, adhesion and viability

**Figure 1:**
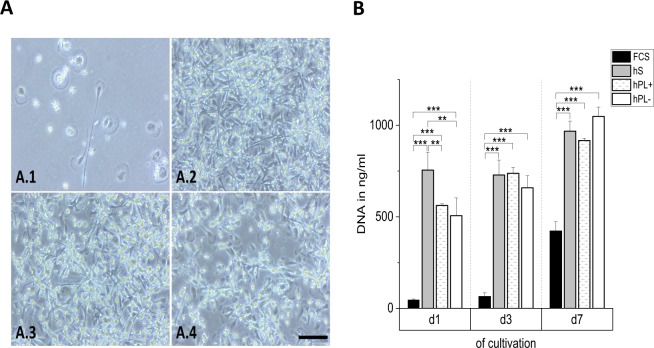
Phenotype and adhesion of spontaneously differentiated macrophages. The phenotype (**A**) of macrophages after seven days of cultivation in medium with hS (A.2), hPL+ heparin (A.3), and hPL− heparin (A.4) was similar, while macrophages cultivated in medium with FCS showed a markedly different morphology (A.1). The analysis of DNA amounts (**B**) corresponding to cell numbers, provided information about cell adhesion behavior (mean ± SD, n = 3). Scale bar: 100 µm. **p < 0.01, ***p < 0.001.

Possible differences regarding the maturation and phenotype characteristics of monocytes/macrophages after cultivation in RPMI-1640 medium supplemented with 10% hS, 10% FCS, and 10% hPL+/− heparin, respectively, were analyzed by inverted light microscopy after a culture period of seven days (Fig. 1A). After maturation, several macrophages in medium with hS (Fig. 1A.2) showed a round shape with a diameter size of approximately 20 µm. In addition, cells with long cellular extensions were observed in this media condition. Macrophages in media supplemented with hPL+/− heparin showed similar morphologies compared to hS (Fig. 1A.3,4). In contrast, the cultivation of macrophages in medium with FCS (Fig. 1A.1) yielded a markedly different phenotype with flattened cells with a diameter of up to 50 µm. The difference in the cell numbers of macrophages in hS/hPL compared to macrophages cultivated with FCS was quantified via the measurement of the DNA amounts after one, three and seven days of culture (Fig. 1B). The DNA amount and hence cell numbers increased over time from day one to day seven of cultivation in all culture media. Since macrophages do not proliferate *in vitro*, higher cell numbers reflect a stronger cell adhesion to the plastic surface with time. Thereby, in media supplemented with hS and hPL+/− heparin, cell adhesion was at least 2-fold (d7, approx. 1 µg/ml DNA) elevated compared to that in medium with FCS (0.42 µg/ml DNA). Cell viability was not affected as indirectly analyzed via flow cytometry for the apoptotic cell marker 7AAD (Fig. S1, Supporting Information).

#### Gene and protein expression of spontaneous/non-induced and induced differentiated macrophages in reply to different sera and supplements

**Figure 2:**
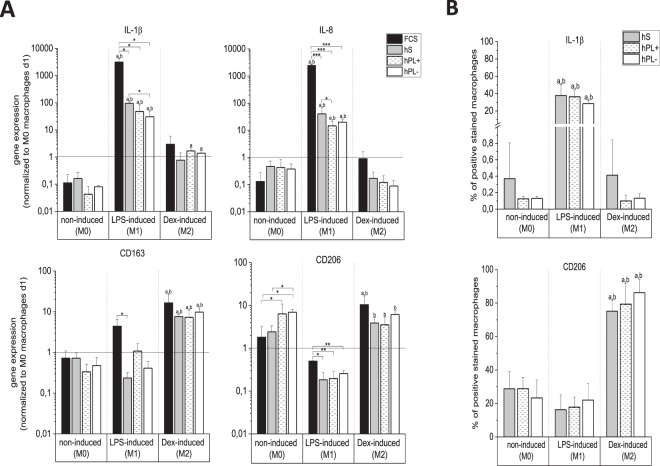
Gene and protein expression of spontaneously (M0) and induced (M1/M2) differentiated macrophages after seven days. (**A**) Gene expression was analyzed by qPCR. Independent of culture conditions, spontaneous as well as induced differentiation was observed. The M1 markers IL-1β and IL-8 were downregulated in the non-induced (M0) as well as Dex-induced (M2) macrophages and highly upregulated in LPS-induced (M1) ones. The M2 markers CD163 and CD206 were more highly expressed in M2 macrophages, compared to M0 and M1. Spontaneous differentiation (M0) differed from the reference sample (monocytes/macrophages on day 1) in all four media (mean ± SD, n = 3). (**B**) Polarization into M0/M1/M2 macrophages determined via protein analysis using flow cytometry. M1 macrophages reflected a 40% higher IL-1β expression compared to M0 and M2. In contrast, M2 macrophages markedly showed a 60% higher protein expression of CD206 in comparison to M0 an M1 macrophages (mean ± SD, n = 3). *p < 0.05; **p < 0.01, ***p < 0.001; ^a^p < 0.05 vs non-induced (M0), same serum supplement; ^b^p < 0.05 between LPS-induced (M1)/Dex-induced (M2), same serum supplement.

Gene expression analysis was performed after seven days of cultivation of spontaneous, non-induced (M0) and lipopolysaccharide (LPS)/dexamethasone (Dex)- induced (M1/M2) macrophages in media supplemented with either hS, hPL+/− heparin or FCS (Fig. 2A) via qPCR. According to the gene expression patterns of IL-1β, IL-8, CD206 and CD163, we were able to show that non-induced (M0) macrophages differentiate spontaneously in all media. Compared to the reference sample of macrophages on day one (expression level set to 1) the expression of IL-1β and IL-8 decreased while the expression of CD206 increased. In M0 cell cultures with hPL, spontaneously differentiated macrophages showed differences in M2 marker expression to those cultivated in hS- and FCS- supplemented cultures. In particular, a decrease of CD163 and an increase of CD206 expression was observed for hPL-supplemented cultures. Induced differentiated macrophages were able to differentiate into both subtypes in all culture conditions. An upregulation of M1 markers and a downregulation of M2 markers in induced M1 differentiated macrophages, and vice versa for M2 differentiated cells compared to spontaneously differentiated ones were detectable. Interestingly, macrophages cultured in medium supplemented with hPL reflect a more similar expression pattern to cells in hS- than in FCS-containing media. The addition of heparin showed no significant differences.

For further analysis of the M0, M1 and M2 polarization, the protein expression of IL-1β and CD206 was determined via flow cytometry after seven days of cultivation (Fig. 2B). In hS as well as in hPL+/− heparin, differentiation into the M1 and M2 type macrophages were confirmed. Accordingly, for M1 macrophages, the M1 marker expression of IL-1β increased significantly up to 37-fold compared to M0 and M2 type macrophages. For the M2 type macrophages, the specific M2 marker CD206 was expressed by 80% of the cells. This corresponds to a 3- (M0) and 5-fold (M1) increase, respectively, compared to the other macrophage types. Thereby, the tested serum supplements did not result in significant differences of marker gene expression within the same differentiation condition. Flow cytometry of macrophages in FCS-supplemented media failed due to low cell numbers adhering onto the well plates and was therefore omitted in this assay (scatter plots shown in Supporting Information, Fig. S2).

#### Cytokine release profile of non-induced (M0) macrophages

**Figure 3:**
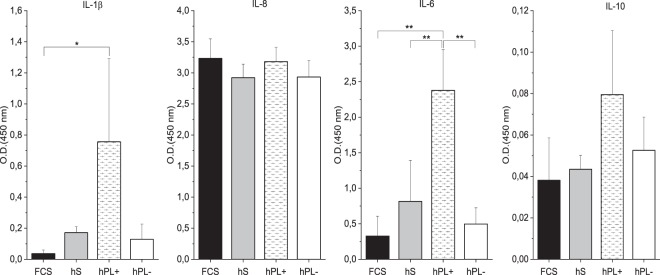
Cytokine release profiles of spontaneously differentiated macrophages after seven days of culture. Cytokine release of IL-1β, IL-6, IL-8, and IL-10 was detected in supernatants of macrophages cultivated in medium with hS, FCS, and hPL+/− heparin using a commercially available ELSIA kit. Relative protein amounts are given by arbitrary units of optical density at 450 nm and show varying release profiles for different sera supplementations (mean ± SD, n = 3). *p < 0.05; **p < 0.01.

The cytokine release, tested for 12 inflammatory cyto- and chemokines, detected four markers (pro-inflammatory: IL-1β, IL-8, IL-6; anti-inflammatory: IL-10) with expression values above the detection limit of the assay (Fig. 3). Thereby, the expression profile of each cytokine varied for different sera supplementation. The release of IL-1β was downregulated in macrophages cultivated in medium with FCS, compared to those cultivated in medium with hS and hPL+/− heparin. The highest expression of IL-6 was observed in the hPL+ heparin group, whereas the lowest level was detectable in FCS. IL-8 was highly expressed in all tested culture conditions. The anti-inflammatory-related cytokine IL-10 was generally released at lower levels with the highest values for macrophages in media with hPL+ heparin.

### Co-culture experiments with monocyte-derived M0 macrophages and hMSCs

#### Phenotype of direct co-culture and quantification of macrophage amount

**Figure 4:**
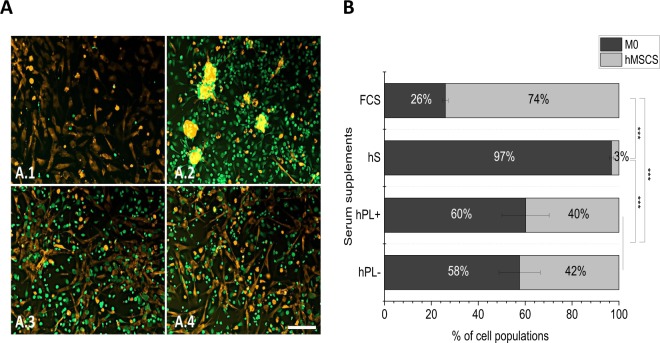
Phenotype and proportion of cell populations in the co-culture of M0 macrophages and hMSCs. The phenotypes of macrophages (green) and hMSCs (orange) were investigated under different culture conditions (**A**). In medium with FCS (A.1) only very few macrophages adhered, in medium with hS (A.2) hMSCs aggregated. In cell cultures with hPL+(A.3)/−(A.4) heparin, both, macrophages and hMSCs, displayed cell type-specific phenotypes corresponding to their morphology in single cultures with the respective standard serum. The percentage of macrophages in co-cultures measured via flow cytometric analysis of CD45 expression (**B**) confirmed the microscopic observations (mean ± SD, n = 4). Scale bar = 200 µm. ***p < 0.001.

For the visualization of the phenotype of both cell types in direct co-culture, macrophages were stained with a green- and hMSCs with an orange-fluorescent non-transferable live cell tracker and imaged via inverted fluorescence microscopy after 72 h of co-cultivation (Fig. 4A). The cultivation in medium with hS led to an accumulation of hMSCs, whereas macrophage adhesion in medium with FCS was reduced. In medium supplemented with hPL+/− heparin instead, macrophages and hMSCs were successfully co-cultivated for the whole culture period of 72 h without any loss of normal cell behavior. The percentage of CD45-positive macrophages under the different culture conditions was determined (Fig. 4B). In co-cultures with hS, almost only macrophages and a small number of hMSC (3%) were detected. In media with FCS in contrast, only 26% macrophages were determined and hence outnumbered by more than 74% of hMSCs. After 72 h of co-cultivation in media with hPL+/− heparin, 60% of CD45-positive macrophages indicated 40% of hMSCs.

#### Gene expression profile of M0 macrophages after co-cultivation with hMSCs

**Figure 5:**
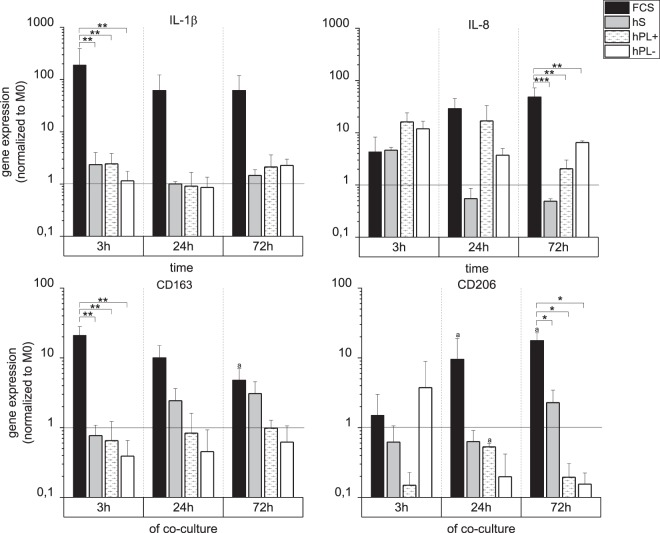
Gene expression profile of M0 macrophages after co-cultivation with hMSCs. The gene expression of the M1 markers IL-1β and IL-8 as well as the M2 markers CD163 and CD206 was analyzed for CD45-positive macrophages after 3 h, 24 h and 72 h of co-cultivation by qPCR. For the different culture conditions (medium supplemented with hS/FCS/hPL+/−), expression levels are depicted with normalization to RPS27A and M0 macrophages (mean ± SD, n = 3). *p < 0.05; **p < 0.01; ***p < 0.001; ^a^p < 0.05 vs 3 h, same serum supplement.

The gene expression profile of macrophages co-cultured for up to 72 h with hMSCs in direct contact was analyzed by qPCR after separation via a leukocyte-specific anti-CD45 magnetic bead system (Fig. 5). Under different culture-conditions (hS, FCS, hPL+/− heparin), highly significant differences were detected. In macrophages cultivated in medium with FCS, IL-1β, IL-8, CD163, and CD206 were upregulated compared to the reference sample (mono-cultivated macrophages (M0)) as well as to macrophages cultivated in medium with other serum types. After 72 h these macrophages showed a significant increase of CD206 and decrease of CD163. During three days of co-cultivation, macrophages in hS displayed a decreased expression of the M1 markers IL-1β and IL-8 as well as an increase of the M2 markers CD163 and CD206. Co-cultivated macrophages in medium with hPL+/− heparin changed their expression pattern over the culture time with a minor upregulation of IL-1β and IL-8 and a downregulation of CD206 compared to the reference sample. Comparing the use of hPL with or without heparin, no significant difference was observed.

#### Phagocytic activity of macrophages after co-cultivation with hMSCs

**Figure 6:**
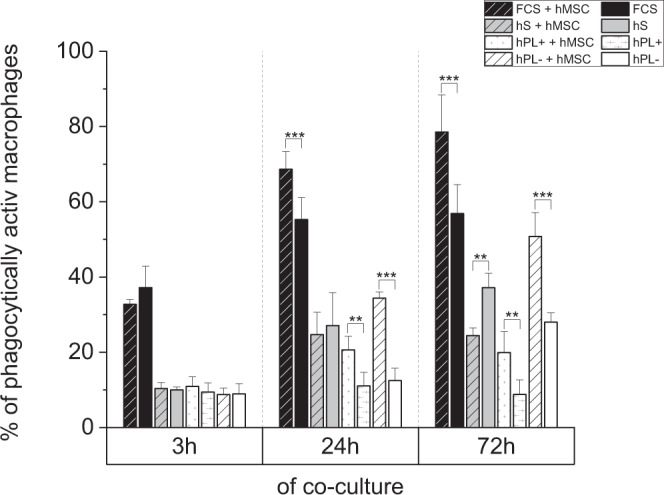
Phagocytic activity of M0 macrophages mono- and co-cultured with hMSCs. The phagocytic activity of CD45-positive macrophages under different culture conditions (medium with hS/FCS/hPL+ and − heparin) was measured via flow cytometry for the uptake of fluorescent beads after 3 h, 24 h, and 72 h. In medium with FCS and hPL, the phagocytosis rate of co-cultured cells exceeded that of the mono-cultured ones. Contrarily, in medium with hS, mono-cultured macrophages even exhibited a higher bead uptake than those in co-culture (mean ± SD, n = 3). **p < 0.01; ***p < 0.001.

The phagocytic activity of macrophages in mono- and co-cultures was measured over a time period of 72 h via flow cytometry for phagocytosed green-fluorescent latex beads and CD45 marker staining to discriminate macrophages from hMSCs (Fig. 6). After 3 h of co-cultivation with hMSCs, macrophages had a phagocytic rate similar to that of mono-cultured cells in all different culture media (supplemented with hS/FCS/hPL+/− heparin). After 24 h and 72 h, the phagocytic activity of co-cultured macrophages increased compared to mono-cultures in medium with FCS and hPL. Only for co-cultured macrophages in medium with hS, phagocytosis was not enhanced and the mono-cultured ones even showed a higher uptake of beads. Cells cultured with FCS showed the highest uptake (up to 70% of green fluorescent cells) at all time points. After 72 h in hPL-supplemented medium, an effect of heparin was detected with a higher phagocytic uptake in the absence of heparin.

## Discussion

In the present study, we demonstrated that human platelet lysate (hPL) is suitable as a serum supplement for monocyte-derived macrophage cultivation, comparable to human serum (hS) and preferable to fetal calf serum (FCS) as media supplements. The macrophage phenotype (Fig. 1A) was conserved in medium with hS and hPL and resembled that of spontaneously differentiated M0 macrophages. In accordance with literature, macrophages were characterized by an elongated as well as roundish shape of approximately 20 µm cell diameter suggesting a spontaneously differentiated phenotype^7^. In contrast, in medium with FCS, macrophages were larger in diameter (up to 50 µm) but less numbers of cells adhered. The latter corresponded to low DNA retrieval from these samples (Fig. 1B). After seven days, the same level of DNA amounts was measured in cell cultures with hS and hPL, while for the supplementation with FCS only half of the previously detected amount was determined. The observation of a DNA increase over time did not arise from cellular amplification, since monocyte-derived macrophages are not able to proliferate *in vitro*. Rather the lack of media changes within the seven-day period analyzed here allowed for more and more still non-adherent macrophages settling down and therefore being included in the analysis. Moreover, the cell viability, indirectly analyzed via the detection of the apoptotic cell marker 7AAD (Fig. S1), was not affected significantly by any of the used culture conditions. The macrophage population included only a small apoptotic cell proportion of approximately 5–7%. Since the DNA amount and hence the cell numbers did not decrease over time (Fig. 1), we excluded the loss of dead cells that might have had detached from the culture plate surface and thus considered the low apoptotic value reliable.

Macrophages are highly heterogenic cells and differentiate upon stimulation by various factors. Their differentiation capacity into the two subtypes M1 and M2, induced by lipopolysaccharides and dexamethasone, respectively, was checked during cultivation with the different serum supplements and yielded the expected subtype characteristics on the gene (Fig. 2A) and protein (Fig. 2B) level. In accordance with the literature^4,5^, the M1-induced macrophages upregulated the M1 markers IL-1β and IL-8 and downregulated the M2 markers CD163 and CD206 while M2-induced macrophages showed the reversed expression pattern. Moreover, the ability of macrophages to differentiate spontaneously into the M0 type by environmental factors is important to investigate. Our study proved that all four culture conditions applied maintained this ability. However, differences in the expression profile on day seven, compared to the reference sample of day one, were observed. While hS and hPL supplementation yield a similar outcome, both are different from the results obtained upon FCS supplementation. This might be explained by the higher similarity of hS and hPL being both human materials, but also the less effective adherence of macrophages in FCS (Fig. 1B) might play a role.

A range of cytokines, which confer instructions and mediate communication among immune cells, plays a central role in the involvement of macrophages in immunity^36^. Hence, we analyzed the effects of the different serum types on the cytokine release of spontaneously differentiated macrophages (Fig. 3). Macrophages in media with hS and hPL without the addition of heparin had a similar cytokine release profile. Additionally, macrophages in medium containing FCS showed no significant differences to the other serum types without heparin. Only macrophages cultivated with hPL plus heparin released significantly higher amounts of the cytokines IL-1β and IL-6 into the cell culture media. We assume that heparin has a stimulating effect on the cytokine release of macrophages. This is in accordance with previous studies that have already described this effect^34^.

To further evaluate the effects of hPL on hMSCs, we have also analyzed their phenotype (Fig. S3) and their differentiation potential in media with either 10% FCS or 5% hPL including 2 U/ml heparin (hPL+) on the protein level via Western blot analyses (Fig. S4). Over the cultivation period of 3 days, hPL+/− supplementation maintained the typical spindle-shaped, fibroblast-like phenotype of hMSCs as observed under standard cultivation with FCS. In contrast, hS supplementation resulted in low cell numbers and star-shaped morphology. The osteogenic marker protein expression (ALPL, BSP, and SPP1; Fig. S4B–D) increased during the time course of osteogenic induction and was even accelerated by hPL+ compared to FCS. Although the amounts of the more general ECM component COL1 diminished over time, its initially higher production in media with hPL+ compared to FCS supplementation might aid the structural support for later mineralization of the osteogenic matrix. Taken together, these data suggest that hPL+ efficiently allows for osteogenesis of hMSCs as has also been reported in literature^37^. Whereas we found heparin to be dispensible within the hPL- supplemented cultivation of macrophages, we still used its supplementation in the differentiation media for MSCs to prevent media gelation. Unlike the studies mentioned above, hMSCs employed in our study were all expanded in FCS-supplemented medium, which excluded the enrichment of different hMSC subpopulations by different sera prior to the initiation of differentiation. With having shown a positive effect of hPL+ on osteogenic differentiation in our study compared to FCS, this may aid in tissue engineering for the treatment of bone loss disorders like osteoporosis, where the acceleration of osteogenesis is needed.

Besides the mono-cultivation of macrophages and hMSCs, we investigated the influence of the different serum supplements on the co-culture with hMSCs putting the main attention to macrophages. The evaluation of the macrophage and hMSC phenotypes within the co-cultures (Fig. 4A) lead to the conclusion that hPL was the best option, since the phenotypes of both cell types corresponded to that of their respective standard culture medium. In contrast, the other media created disadvantages for one or the other cell type. In particular, the cultivation in medium with hS led to the aggregation of hMSCs and media with FCS only allowed for the adhesion of few macrophages to the well plate, as already observed for mono-cultures (Fig. 1B). Accordingly, after 72 h of co-cultivation in medium with hPL, 60% macrophages and hence 40% hMSCs were present (Fig. 4B). In medium with hS, nearly no hMSCs were detectable and in medium with FCS the macrophage proportion was reduced to 26%. Since hMSCs in the samples with hS supplementation were not able to adhere properly and rather formed cell aggregates (Fig. 4A.1), these cells were possibly lost during the sample preparation. Macrophages and hMSCs were cultivated together with a starting ratio of 1 hMSCs to 4 macrophages. Over 72 h of co-cultivation in media with hPL+/− heparin this ratio shift to 1 hMSCs to 1.5 macrophages. Thus, hMSCs were able to proliferate during this time.

The gene expression profiles of macrophages caused by co-cultivation, led to different phenotypes depending on the culture conditions (Fig. 5). As expected, reduced numbers of hMSCs in hS and low cell density of macrophages in FCS affected the gene expression. In hS medium, macrophages developed an M2-like phenotype over the culture period with increased CD206 and CD163, as well as decreased IL-1b and IL-8 expression, compared to the M0 macrophages. In medium containing FCS, no tendency to M1 or M2 phenotype could be detected. Both, M1 (IL-1b, IL8) and M2 (CD163 and CD206) markers showed a higher expression than in the reference samples. Over the period of the experiment there was a significant increase of the CD206 expression and decrease of CD163. When cultivated with hPL and thus in a balanced co-culture, macrophages showed increased IL-8 and decreased CD206 expression and therefore rather an M1-like type. This could be explained by the recognition of foreign cells. Foreign bodies always trigger an inflammatory immune response^38^. Since over the duration of the co-cultivation hardly any hMSCs are present in medium with hS and likewise most of the macrophages were absent in FCS. Therefore, some differences in the expression patterns were expected. When compared to serum types, significant differences were found only in relation to FCS. We hypothesize that this is due to the significantly decreased macrophage population in medium with FCS. It might be necessary for the cells to express higher levels of marker in order to share the information and cell responses on hMSCs with neighboring cells.

Phagocytosis of pathogens or foreign bodies is one of the main characteristics of macrophages. It is known that hMSCs can enhance the phagocytic activity in co-culture systems^39,40^. Regarding this phenomenon the phagocytic activity of macrophages in co-culture was compared to those in mono-culture (Fig. 6). Co-cultures in medium with FCS and hPL yielded a higher phagocytic rate than single cultures. Contrarily, co-cultured macrophages in medium with hS showed a lower phagocytosis rate than mono-cultured ones. Hence, the presence of hMSCs in media preserving this cell type (i.e. media with FCS and hPL) supports and ameliorates bead uptake which is in accordance with the known ability of hMSCs to enhance phagocytosis.

Co-cultures of different cell types are important for a better understanding of cellular interactions *in vitro*. However, their establishment is often difficult, because each cell type is usually cultivated under different conditions^41^. Thus, co-cultivation is commonly a compromise to preserve the most important cell properties of both cell types. Experiments performed in media supplemented with hPL (without the use of heparin) showed both, macrophages and hMSCs, have comparable phenotypes to their standard cultivation sera. Furthermore, both cell types retain their property to differentiate, spontaneously (macrophages) as well as induced (macrophages, hMSCs). In the direct co-culture, macrophages and hMSCs were cultivated together without disadvantages, i.e. the maintenance of hMSCs could be shown and the enhancement of phagocytosis by macrophages was preserved. Neither cultivation with hS nor FCS could achieve this totally.

In all experiments performed here, the need for heparin during the culture of macrophages with hPL was tested. Neither cell shape, gene expression, nor phagocytic activity showed a significant difference between hPL with or without heparin. However, significantly differences on the cytokine profile could be detected in medium containing heparin compared to all other sera. Furthermore, despite the omission of heparin in our hPL- samples, no gelation of the medium was visible as had contradictorily been described in previous studies^33^. Hence, this suggests that the expression of heparan sulfate proteoglycans by monocytes and macrophages themselves, which has been described before^42^, was as sufficient to act as an anticoagulant factor as exogenous heparin^43^. We therefore recommend avoiding additional heparin during macrophage culture, especially because of higher amounts of released proinflammatory cytokines IL-1β and IL-6 (Fig. 3) and other possible stimulation side effects on macrophages^34,35^. Moreover, hMSCs in co-cultures might be influenced by higher anticoagulant concentrations, resulting in impaired cellular proliferation and reduced colony-forming units^33^.

Despite some studies employed FCS for the culture of human macrophages^44–46^, we strongly advise not to do so, based on the results stated in this study and due to ethical considerations.

## Conclusion

This study demonstrates that hPL, in particular without adding heparin, can efficiently replace hS for the *in vitro* culture of human macrophages without any restrictions. In co-culture experiments of primary human macrophages and hMSC; we show clear negative effects of FCS as culture supplement. Using hPL as best performing serum supplement, we were able to define a co-culture system for human macrophages and hMSCs without the need for heparin in the culture medium. We envision that this system will be of great value for research questions that imply co-culture of macrophage and hMSCs, with biomaterial assessment under culture conditions that more closely mimic the early phase of body response after implantation as one important example.

## Material and Methods

### Cell culture

All Experiments were performed with the approval of the Local Ethics Committee of the University of Würzburg. Buffy-Coats were obtained from the Bavarian Red Cross with a written informed consent of each blood donor.

Monocytes were isolated from human blood-derived buffy coats (Blood donor service, German Red Cross, Wiesentheid (D)) of healthy donors. Peripheral blood mononuclear cells were collected by density gradient centrifugation with Pancoll (Density: 1,077 g/l; Pan-Biotech, Aidenbach (D)). Monocytes were then isolated via negative selection (Pan Monocyte Isolation Kit, Miltenyi Biotec, Gladbach (D)) and cultivated up to seven days in macrophage culture medium (RPMI-1640, GlutaMAX^TM^ medium (Thermo Fischer Scientific, Waltham (USA)) with either 10% of human serum (hS, pooled serum of 6 healthy donors), 10% fetal calf serum (FCS, Thermo Fisher Scientific, Waltham (USA)) or 10% of human platelet lysate (hPL, PL Bioscience, Aachen (D)) with (+) or without (−) addition of 2 units/ml of heparin (PL Bioscience, Aachen (D)) and 1% Penicillin-Streptomycin (Pen-Strep; 5,000 U/mL) (Thermo Fisher Scientific, Waltham (USA)) in non-treated 12-well plates (Corning, Corning (USA)) in a humidified atmosphere at 37 °C and 5% CO_2_ without any medium change. Monocytes differentiated spontaneously, i.e. without supplemented differentiation factors, into macrophages within this time (M0 macrophages).

For the induced differentiation of monocytes into M1 and M2 type macrophages, respectively, 1 µg/ml lipopolysaccharides (Sigma-Aldrich, Munich (D), M1) and 10^−7^ M dexamethasone (Sigma-Aldrich, Munich (D), M2) were used.

HMSCs from trabecular bone were isolated from the femoral heads of patients undergoing total hip arthroplasty and selected via plastic adherence^47^. All experiments were approved by the Local Ethics Committee of the University of Wuerzburg with the written informed consent of each donor patient. HMSCs were routinely tested for their differentiation potential along the adipose-, chondrogenic- and osteogenic lineage. Obtained cells were cultured in DMEM F-12 medium (Thermo Fisher Scientific, Waltham (USA)) with 10% FCS, 1% Pen-Strep and 50 µg/ml L-ascorbic-acid-2-phosphate (Sigma Aldrich, Munich (D)) in 175 cm^2^ cell culture flasks (Greiner Bio-One, Kremsmuenster (D)). Medium was changed every three to four days.

Cell passaging was performed at 90% confluence. For all experiments, undifferentiated hMSCs in passage 2 were used.

### Co-culture experiments

For co-culture experiments, monocytes/macrophages were cultivated four days on suspension plates (Sarstedt, Nürnbrecht (D)). After that time, hMSCs in a ratio of one (hMSCs) to four (macrophages) in passage 2 were added onto the monocytes/macrophages in fresh macrophage culture medium. Co-culture studies were performed for up to three days. For gene expression analysis, macrophages were separated after co-cultivation via magnetic CD45 MicroBeads (Miltenyi Biotec, Bergisch Gladbach (D)) according to the manufacturer’s protocol.

### Inverted light and fluorescence microscopy

The cell shape of macrophages was monitored via inverted light microscopy. For the discrimination of both cell types in the co-culture, cells were stained with 50 nM non-transferable CellTracker^TM^ fluorescent dyes (Thermo Fisher, Invitrogen) (macrophages: green CMFDA; hMSCs: orange CMRA). Specimens were analyzed via fluorescence microscopy (Axio Observer, Zeiss, Oberkochen (D), equipped with epifluorescence optics and a XY camera).

### Determination of DNA amount

To determine the DNA amount and thereby the cell adhesion of macrophages, the Quant-iT™ PicoGreen® dsDNA Reagent and Kit (Thermo Fisher Scientific, Waltham (USA)) was used per the manufacturer’s manual. In short, macrophages were cultivated in a 24-well plate in 1 ml macrophage culture medium in a humidified atmosphere at 37 °C and 5% CO_2_. After one, three and seven days, cells were washed once with PBS^−^ and lyzed in 1 ml 1% Tritonx-100 in PBS^−^ for 1 hour at 4 °C. The standard curve was prepared as described for the middle-range in the manual. The samples were excited at 485 nm and the fluorescence emission intensity was measured at 538 nm on a plate reader (Tecan, Männedorf (CH)).

### Gene expression analysis

Total cellular RNA of macrophages was isolated using PeqGold Trifast (VWR, Darmstadt (D)) per the manufacturer’s protocol. Afterwards cDNA was generated with the High-Capacity cDNA Reverse Transcription Kit (Thermo Fisher Scientific, Waltham (USA)) according to the manufacturer’s manual. The mRNA levels of macrophages were analyzed via quantitative Real-Time PCR (qPCR) (StepOnePlus; Thermo Fisher Scientific, Waltham (USA)) with “Sybr Select” Mastermix (Thermo Fisher Scientific, Waltham (USA)). For the amplification of the mRNA, each 10 µl qPCR reaction comprised 5 ng of cDNA and 200 nM primer sequences (Biomers, Ulm (D)) (Table 1). For each cDNA sample, the threshold cycle (Ct) value of each target sequence was subtracted from the Ct value of the house keeping mRNA RPS27a, to derive ΔCt. The RQ values were calculated by the 2^−ΔΔCt^ method. A control group of macrophages was used for normalization.

**Table 1:** Primer sequences.

Name	Sequence 5′ → 3′	Annealing temperature [°C]	Fragment size [bp]
**RPS27A**			141
Forward	5′-TCGTGGTGGTGCTAAGAAAA-3′	61	
Reverse	5′-TCTCGACGAAGGCGACTAAT-3′		
**IL-8**			113
Forward	5′-CATACTCCAAACCTTTCCACCC-3′	61	
Reverse	5′-CTCTGCACCCAGTTTTCCTTG-3′		
**IL-1β**			120
Forward	5′-GACCTGAGCACCTTCTTTCCC-3′	61	
Reverse	5′-GCACATAAGCCTCGTTATCCC-3′		
**CD163**			85
Forward	5′-GTGCCTGTTTTGTCACCAGTTC-3′	61	
Reverse	5′-TTACACACCGTTCCCCACTCC-3′		
**CD206**			156
Forward	5′-TCCAAACGCCTTCATTTGCC-3′	61	
Reverse	5′-GCTTTTCGTGCCTCTTGCC-3′		

### Flow cytometry

The surface proteins CD45 and CD206 as well as the intracellular protein IL-1β were quantified via flow cytometric analysis on a FACSCalibur™ device (BD Bioscience, Heidelberg (D)). For this, cultured macrophages were scraped off the well bottom and incubated with specific antibodies and the corresponding non-immune isotype control, respectively, according to manufacturer’s protocol (Table 2). For the staining of intracellular IL-1β, samples were prepared with the “InsideStain Kit” (Miltenyi Biotec, Bergisch Gladbach (D)). After centrifugation at 300 × *g* for 10 min, the supernatant was discarded and the pellets were resuspended with 500 µl FC-buffer (phosphate-buffered saline (PBS), pH 7.2, 0.5% bovine serum albumin (BSA), 2 mM EDTA). Data was analyzed with the Software “FlowJo” (FlowJo LLC, Ashland (USA)). Appropriate cell gating excluded dead cells and cell debris.

**Table 2:** Antibodies for flow cytometry.

Antibody against	Isotype	Fluorescence dye	company
CD206, human	Mouse IgG1	FITC	Biolegend, San Diego (USA)
CD45, human	recombinant human IgG1	FITC	Miltenyi Biotec, Bergisch Gladbach (D)
IL-1β, human	Mouse IgG1	APC	Biolegend

### Cytokine quantification via Multi-Analyte ELISArray

Cytokine release of spontaneously differentiated macrophages was tested via the Multi-Analyte ELISArray Kit (Qiagen, Hilden (D)) after seven days of cultivation. The production of IL-1α, IL-1β, IL-2, IL-4, IL-6, IL-8, IL-10, IL-12, IL-17A, IFN-γ, TNF-α and GMCSF was analyzed in supernatants according to the manufacturer’s protocol. The absorbance was measured on a plate reader (Tecan, Männedorf (CH)) at 450 nm, corrected with the absorbance at the reference wavelength of 570 nm.

### Phagocytosis assay

The phagocytic activity of macrophages was analyzed using 2 µm green fluorescence-labeled latex beads (Sigma-Aldrich, Munich (D)). The beads were opsonized in pooled human serum for 30 min prior to addition to the macrophage culture in a ratio of 10 beads per seeded cell and incubated in a humidified atmosphere for two h at 37 °C and 5% CO_2_. Cells were washed three times with serum-free macrophage culture medium to remove non-phagocytosed beads.

Bead uptake was analyzed via flow cytometry. Therefore, macrophages were scraped off the well plate, transferred into suitable 5 ml tubes (Sarstedt, Nümbrecht (D)), and centrifuged (300 × *g*, 5 min, 4 °C). The supernatant containing free-floating beads was discarded, whereas the pellet was resuspended in 500 µl of FC-buffer and the number of positive cells was quantified. For the discrimination of cell types in co-culture studies with hMSCs, cells were stained against leukocyte-specific CD45 (Milteny Biotec; labeled with APC) according to the manufacturer’s instruction. Double-positive cells were determined as bead-containing macrophages.

### Statistics

Statistica (StatSoft, Tulsa, USA) was used for statistical analyses. The statistical significance of qPCR data was determined by two-sample t-test, for all other data, two-way analysis of variance (Anova) was performed. Results were considered to be significantly different at a p value below 0.05.

### Informed consent

Informed consent has been obtained from all individuals included in this study.

### Ethical approval

The research related to human use has been complied with all the relevant national regulations, institutional policies and in accordance with the tenets of the Helsinki Declaration, and has been approved by the authors’ institutional review board or equivalent committee.

## Supplementary information

supplementary information
