# Supplementary material for: Platelet lysate outperforms FCS and human serum for co-culture of primary human macrophages and hMSCs
Source: Sci Rep. 2019 Mar 5;9:3533. doi: 10.1038/s41598-019-40190-9 (PMC6401182; doi:10.1038/s41598-019-40190-9)
Supplement: Supplementary file 1 — supplementary information [file 41598_2019_40190_MOESM1_ESM.docx]

**Supporting Information to**

**Platelet lysate outperforms FCS and human serum for co-culture of primary human macrophages and hMSCs**

Tina Tylek^1^, Tatjana Schilling^1^, Katrin Schlegelmilch^1^, Maximilian Ries^1^, Maximilian Rudert^2^, Franz Jakob^2^, Jürgen Groll^1*^

1. Department for Functional Materials in Medicine and Dentistry and Bavarian Polymer Institute, University of Würzburg, Würzburg, Germany.
2. Department of Orthopedics, Orthopedic Center for Musculoskeletal Research, University of Würzburg, Würzburg, Germany

**Material and Methods**

**Quantification of apoptotic cells**

7-amino-actinomycin D (7AAD)-solution (Miltenyi Biotec, Bergisch Gladbach (D)) was used to distinguish between viable and apoptotic/dead cells according to the manufacturer's instructions. For staining, cells were scraped off the 12-well plate and centrifuged at 300 x *g* for 10 min. Each pellet was resuspended in 500 µl FC-buffer, containing 0.525 µg/mL 7AAD-solution. Samples were analyzed via flow cytometry. No gating was performed, in order not to exclude dead cells. Positive cells were interpreted as apoptotic.

**HMSC differentiation**

For osteogenic differentiation, hMSCs were seeded in propagation media with either 10% FCS or 5% human platelet lysate plus 2 U/ml heparin (hPL+). When the monolayers had reached confluence (d 0), differentiation was induced by chemical induction as described before [S1] for up to 21 days while the respective serum supplement was maintained. Total protein samples were collected on d 0, d 7, d 14, and d 21 of differentiation by using peqGOLD TriFast™ reagent according to the manufacturer's instructions (VWR, Darmstadt (D)) with protein reconstitution in 1% SDS. Protein concentration was determined via the Pierce™ BCA Protein Assay Kit (Thermo Fisher, Darmstadt (D)) according to the manufacturer's instructions. Sodium dodecyl sulfate-poly acrylamide gel electrophoresis (SDS-PAGE) was performed with 10 µg protein sample per lane diluted in Lämmli buffer (2% SDS, 0.004% bromophenol blue, 5% glycerol, 5% beta-mercapto ethanol in 62.5 mM Tris HCl, pH 6.8; all Sigma-Aldrich, Munic (D)) using a Mini-PROTEAN Tetra Cell device (Bio-Rad, Munic (D)) with 4% stacking gel and 12% resolving gel according to the manufacturer's instructions. Subsequent transfers to nitrocellulose membranes were performed with a Trans-Blot Turbo Blotting System (Bio-Rad, Munic (D)) according to the manufacturer's instructions. The transferred proteins were probed with primary antibodies against collagen 1 (COL1; #AB758, Merck, Darmstadt (D); 1:5000), alkaline phosphatase liver, bone, kidney (ALPL; #TA307702, Acris Antibodies, Herford (D); 1:5000), bone sialo protein (BSP; #AV36681, Sigma-Aldrich, Munic (D); 1:1000), secreted phosphoprotein 1 (SPP1; # TA307687, Acris Antibodies, Herford (D); 1:1000), and beta-tubulin (#T4026, Sigma-Aldrich, Munic (D); 1:100) respectively, followed by the incubation with appropriate peroxidase-conjugated secondary antibodies (anti-goat IgG, #81-1620, Invitrogen via Thermo Fisher, Darmstadt (D); 1:5000; anti-rabbit IgG #111-035-003, Jackson ImmunoResearch via Dianova, Hamburg (D); 1:5000). Enhanced chemiluminescent (ECL) reagent (0.02 mg/ml luminol, 0.01 mg/ml p-hydroxy coumaric acid, 0.01% H_2_O_2_ in in 0.1 M Tris HCl pH 8.6; all Sigma-Aldrich, Munic (D)) was used for the visualisation of protein bands on X ray films. Digital photographs of the exposed films were subjected to densitometric analysis using the image analysis software Fiji [S2]. Thereby, the house keeping protein amounts of beta-tubulin were used as internal loading control and the d 0 sample of the respective incubation served for normalization.

**Results**


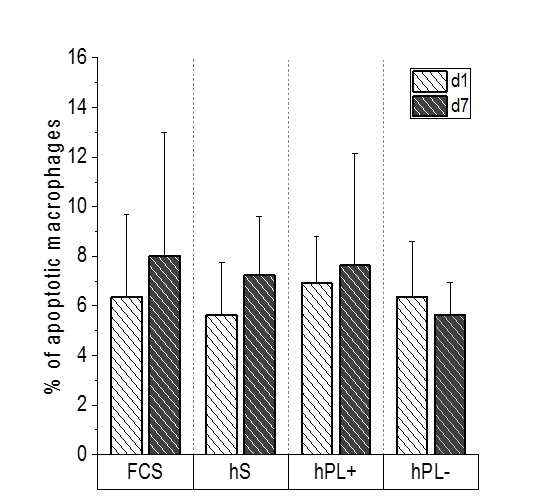


**Figure S1:** **Proportion of apoptotic macrophages in different media conditions.** Positively stained macrophages for the apoptotic cell marker 7AAD were normalized to total cell numbers. The comparison of all four media supplements did not yield significant differences in cell viability (mean ± SD, n = 4).


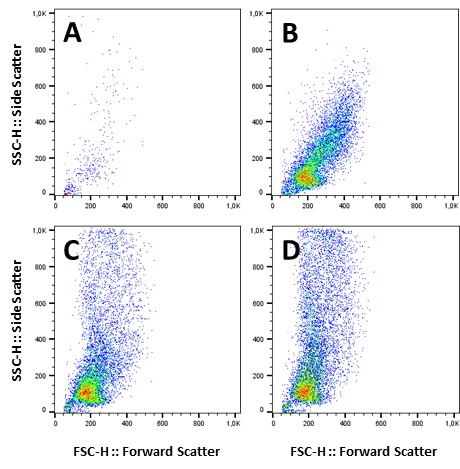


**Figure S2:** **Flow cytometric scatter plots of macrophages cultivated in different sera and stained with specific antibodies.** Due to the lack of macrophages in medium supplemented with FCS (A) flow cytometric analysis was not possible. In Medium with hS (B) as well as hPL+/- heparin (C/D) enough cells for reliable results were present.

**
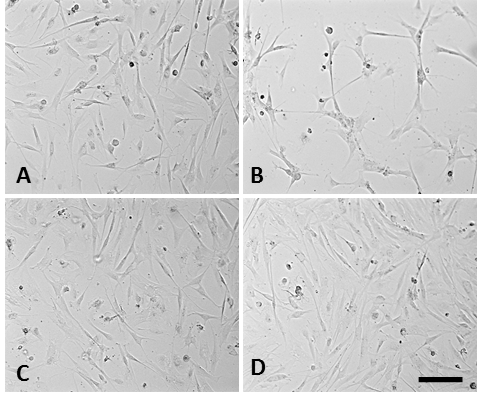
**

**Figure S3: Phenotype of hMSCs in medium supplemented with different sera after 3 days of cultivation.** Via microscopy, differences in phenotype were observed after cultivation in medium supplemented with FCS (A), hS (B), and hPL +/- heparin (C/D). HMSCs in media with FCS, the conventionally used serum, and hPL+/- had a similar, spindle-shaped phenotype while those in hS displayed a different, star-shaped morphology. Scale bar: 100 µm


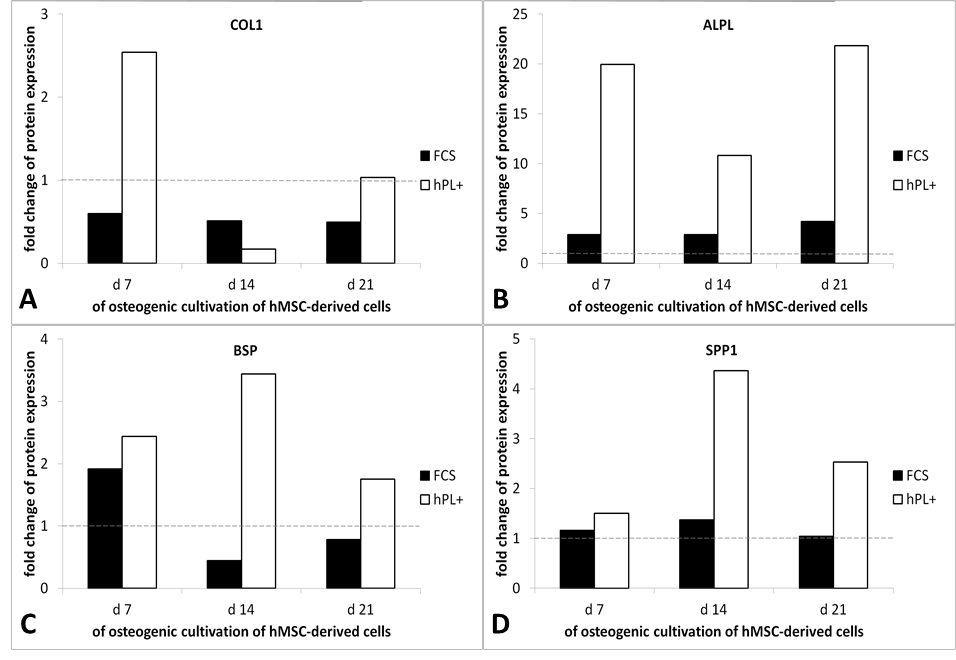


**Figure S4:** **Protein expression changes during the induced differentiation of hMSC-derived cells with different serum supplementation.** Mono-cultures of hMSCs were grown to confluence in media supplemented with 10% FCS and 5% hPL + 2 U/ml heparin (hPL+), respectively. Osteogenic differentiation was induced on d 0 by chemical induction while the respective serum supplement was maintained. Western blot analyses of protein lysates harvested at the indicated time points and on d 0 were performed with specific antibodies against osteogenic (COL1, ALPL, BSP, SPP1) protein markers and normalized to beta-tubulin as internal loading control. Respresentative analyses for one donor are shown. Data were normalized to the respective d 0 sample (dashed line). The early (ALPL) as well as late (BSP, SPP1) osteogenic protein expression was increased upon differentiation and further elevated in media with hPL+ as serum supplement. Although COL1 protein amounts diminished over time, an initially higher production of this more general ECM component in media with hPL+ compared to FCS supplementation was detected.

**References for supporting information**

[S1] T. Schilling, R. Küffner, L. Klein-Hitpass, R. Zimmer, F. Jakob, und N. Schütze, „Microarray analyses of transdifferentiated mesenchymal stem cells“, *J. Cell. Biochem.*, Bd. 103, Nr. 2, S. 413–433, Feb. 2008.

[S2] J. Schindelin *u. a.*, „Fiji: an open-source platform for biological-image analysis“, *Nat. Methods*, Bd. 9, Nr. 7, S. 676–682, Juni 2012.
